# Artificial Intelligence in the Early Prediction of Cardiogenic Shock in Acute Heart Failure or Myocardial Infarction Patients: A Systematic Review and Meta-Analysis

**DOI:** 10.7759/cureus.50395

**Published:** 2023-12-12

**Authors:** Apurva Popat, Sweta Yadav, Sagar K Patel, Sasanka Baddevolu, Susmitha Adusumilli, Nikitha Rao Dasari, Manoj Sundarasetty, Sunethra Anand, Jawahar Sankar, Yugandha G Jagtap

**Affiliations:** 1 Internal Medicine, Marshfield Clinic Health System, Marshfield, USA; 2 Internal Medicine, Gujarat Medical Education & Research Society (GMERS) Medical College, Ahmedabad, IND; 3 Internal Medicine, Gujarat Adani Institute of Medical Sciences, Bhuj, IND; 4 Internal Medicine, Kurnool Medical College, Kurnool, IND; 5 College of Medicine, Chongqing Medical University, Chongqing, CHN; 6 College of Medicine, Kamineni Academy of Medical Sciences and Research Centre, Hyderabad, IND; 7 Radiodiagnosis, Bhaskar Medical College and General Hospital, Hyderabad, IND; 8 Internal Medicine, Chengalpattu Medical College and Hospital, Chennai, IND; 9 Paediatrics, General Medicine, Mahatma Gandhi Mission (MGM) Medical School, Mumbai, IND

**Keywords:** prediction model in medicine, artificial intelligence in medicine, machine learning in medicine, artificial intelligence in cardiology, cardiogenic shock

## Abstract

Cardiogenic shock (CS) may have a negative impact on mortality in patients with heart failure (HF) or acute myocardial infarction (AMI). Early prediction of CS can result in improved survival. Artificial intelligence (AI) through machine learning (ML) models have shown promise in predictive medicine. Here, we conduct a systematic review and meta-analysis to assess the effectiveness of these models in the early prediction of CS. A thorough search of the PubMed, Web of Science, Cochrane, and Scopus databases was conducted from the time of inception until November 2, 2023, to find relevant studies. Our outcomes were area under the curve (AUC), the sensitivity and specificity of the ML model, the accuracy of the ML model, and the predictor variables that had the most impact in predicting CS. Comprehensive Meta-Analysis (CMA) Version 3.0 was used to conduct the meta-analysis. Six studies were considered in our study. The pooled mean AUC was 0.808 (95% confidence interval: 0.727, 0.890). The AUC in the included studies ranged from 0.77 to 0.91. ML models performed well, with accuracy ranging from 0.88 to 0.93 and sensitivity and specificity of 58%-78% and 88%-93%, respectively. Age, blood pressure, heart rate, oxygen saturation, and blood glucose were the most significant variables required by ML models to acquire their outputs. In conclusion, AI has the potential for early prediction of CS, which may lead to a decrease in the high mortality rate associated with it. Future studies are needed to confirm the results.

## Introduction and background

Despite the advances in diagnosis and treatment, 10% to 30% of hospitalized patients due to acute cardiac disorders, such as acute myocardial infarction (AMI) and heart failure (HF), still experience cardiogenic shock (CS), which results in an unacceptable high death rate [1,2]. CS increases the mortality of patients with AMI or HF eightfold and is associated with a mortality rate of 50% or even higher [3]. Furthermore, heart failure and cardiogenic shock showed a recent increase in their prevalence [4,5]. Therefore, understanding the underlying causes and factors that contribute to high mortality in CS is critical.

CS is characterized by a decline in cardiac function, in addition to rapidly progressing multiorgan dysfunction, severe cellular and metabolic abnormalities, and an intractable vicious cycle that cannot be broken, not even by removing the underlying primary cause [6]. Therefore, one of the factors that are associated with the high mortality in CS is the difficulty in detecting and treating the condition early enough to reverse individuals' negative clinical outcomes.

Early identification of high-risk patients before the development of CS might prevent the development of CS and, in the end, improve the survival rate of patients by implementing preventive measures like the implantation of percutaneous mechanical circulatory support [7]. Early diagnosis, triage, risk classification, and treatment of CS patients have shown the potential to improve the prognosis of the disease in observational studies [8]. However, it can be difficult for clinicians to detect, evaluate, identify, effectively triage, manage, and possibly transfer patients to specialized clinics in a timely manner. As such, there is a large unmet demand for methods to accurately identify those at risk of acquiring CS early.

Artificial intelligence (AI) is the capability of computers and non-human systems to make decisions based on the information they are provided with [9]. Machine learning (ML) is a subfield within AI that describes a system's capacity to learn from more data and perform better when making predictions or decisions when exposed to more data [9]. Machine learning algorithms can perform automated, continuous screening and, therefore, be integrated into standard clinical workflows to provide early warnings. ML algorithms showed the ability to better predict readmission after hospitalization for heart failure compared to other traditional techniques [10]. With the presence of electronic health records (EHRs), such as the MIMIC (Medical Information Mart for Intensive Care) dataset, which is a widely-used public data source including over fifty thousand de-identified EHRs of patients admitted to critical care units [11], the development of ML algorithms based on the routinely recorded clinical variables in these records to predict the development of CS is achievable. This highlights the potential of ML to be useful in the early prediction of CS in patients with HF or AMI. 

The literature on CS prediction focuses on mortality predictors for patients who have already developed CS [12]. However, the effectiveness of ML models in predicting the development of CS has not been systematically evaluated. Here, we conduct the first systematic review and meta-analysis to assess the effect of AI in the early prediction of CS in patients with AMI or HF. Furthermore, we investigated the predictor variables that had the most impact on predicting CS, as identified by these models.

## Review

Methods

The authors followed the PRISMA guidelines for systematic reviews and meta-analyses of randomized controlled trials (RCTs) [13].

Eligibility Criteria

We included studies based on the PICOS criteria: patients, intervention, control, outcomes, and study design. The population of interest was patients who were hospitalized due to acute myocardial infarction or heart failure. The deployment of a predictive ML model on their data for early prediction of CS was considered an intervention. No control was needed in our study. To be considered for inclusion in our review, studies must have measured and reported our outcomes of interest. In line with our aim, we included cohort studies, including retrospective or prospective observational single-arm studies. There were no time restrictions for follow-up. No limitations were placed on the publication's timing, country, or race. We excluded non-English studies. 

Information Sources

A thorough search of the PubMed, Web of Science, Cochrane, and Scopus databases was conducted from the time of inception until November 2, 2023, in order to find relevant studies. We also looked through the eligible papers' reference lists to locate any additional relevant studies.

Search Strategy

The databases were searched using a combination of the following terms: "machine learning", "cardiogenic shock", "heart failure", and "myocardial infarction". No filters were applied. The full search strategy for each database is shown in Table 1. 

**Table 1:** A full search strategy is used in each database

Database	Search terms	Search Field	Search Results
PubMed	(“machine learning” OR “clinical decision support” OR “predictive model” OR “artificial intelligence” OR “deep learning”) AND (“cardiogenic shock” OR "Shock, Cardiogenic"[Mesh]) AND (“heart failure” OR “Cardiac Failure” OR “Myocardial Infarction” OR “Myocardial Infarct” OR “Heart Attack” OR “Heart Attacks” OR “Cardiovascular Stroke”)	All Fields	38
Cochrane	(“machine learning” OR “clinical decision support” OR “predictive model” OR “artificial intelligence” OR “deep learning”) AND (“cardiogenic shock”) AND (“heart failure” OR “Cardiac Failure” OR “Myocardial Infarction” OR “Myocardial Infarct” OR “Heart Attack” OR “Heart Attacks” OR “Cardiovascular Stroke”)	All Fields	2
WOS	(“machine learning” OR “clinical decision support” OR “predictive model” OR “artificial intelligence” OR “deep learning”) AND (“cardiogenic shock”) AND (“heart failure” OR “Cardiac Failure” OR “Myocardial Infarction” OR “Myocardial Infarct” OR “Heart Attack” OR “Heart Attacks” OR “Cardiovascular Stroke”)	All Fields	40
SCOPUS	TITLE-ABS-KEY ((“machine learning” OR “clinical decision support” OR “predictive model” OR “artificial intelligence” OR “deep learning”) AND (“cardiogenic shock”) AND (“heart failure” OR “Cardiac Failure” OR “Myocardial Infarction” OR “Myocardial Infarct” OR “Heart Attack” OR “Heart Attacks” OR “Cardiovascular Stroke”))	Title, Abstract, Keywords	84

Selection Process

All of the records were combined using Endnote. After the data were exported to an Microsoft Excel (Redmond, USA) sheet, the sheet was submitted in two stages in order to identify research that was eligible. Articles that pass the initial title and abstract screening stage advance to the full-text screening stage. Each step was assessed separately by two authors, and any conflicts were resolved through discussion.

Data Collection Process and Data Items

The authors constructed a preformatted Excel sheet containing the outcomes of interest and study characteristics and extracted the data from it. Data from each study were extracted separately by two authors, and differences were resolved through discussion.

Data items (outcomes)

The primary outcome was the area under the curve (AUC), where AUC ranges from 0 to 1. A model with 100% incorrect predictions has an AUC of 0.0; one with 100% correct predictions has an AUC of 1.0 [14].

Secondary outcomes included the predictor variables, which are variables identified by the ML predictive model to be statistically significantly correlated with the occurrence of cardiogenic shock in hospitalized patients due to MI or HF, the sensitivity and specificity of the ML model, and the accuracy of the ML model.

Data Items (Other Variables)

Study characteristics included study ID, country, sample size, study design, population condition, population demographics such as age and gender, CS diagnosis criteria used in the study, and data collection methods used by the studies to collect variables for the predictive model.

Statistical Analysis

Comprehensive Meta-Analysis (CMA) Version 3.0 was used to conduct the meta-analysis [15]. The data were pooled and provided as a mean with a 95% confidence interval (CI). To assess heterogeneity, Cochrane's Q test and the I2 statistic were employed. When the p-value was less than 0.1, the heterogeneity was considered significant, and a random-effects model was employed; otherwise, a fixed-effects model was used. The source of the significant heterogeneity was identified via sensitivity analysis.

Results

Literature Search Results

**Figure 1:**
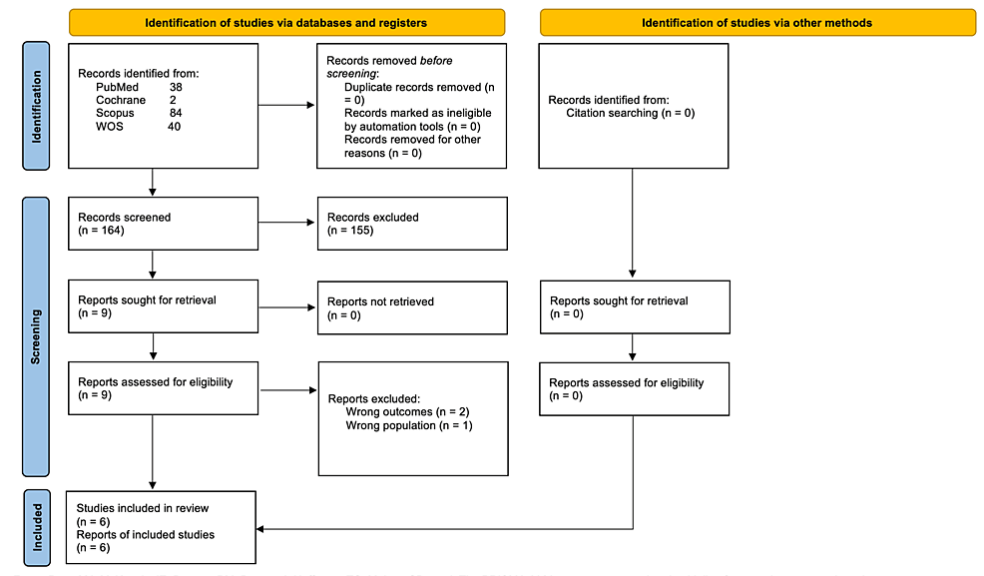
A PRISMA flowchart shows the detailed process of the search strategy and study selection. From: The PRISMA 2020 statement: an updated guideline for reporting systematic reviews. For more information, visit: http://www.prisma-statement.org/

A database search yielded 164 results. Only nine papers were eligible for full-text screening after title and abstract screening. Finally, six studies were determined to be eligible for inclusion in the final analysis. Figure 1 depicts the PRISMA flow diagram.

Study Characteristics

Our systematic review included four retrospective studies [16-19] and two prospective cohort studies [20,21]. All studies were conducted in the USA except for Bai et al. 2021 [21], which was conducted in China. Rahman et al. 2022 [19] included patients hospitalized with acute decompensated heart failure, whereas two studies [16,18] included patients having cardiac catheterizations for acute coronary syndrome. The remaining three studies [17,20,21] included patients from various backgrounds, including those with heart failure and myocardial infarction. All studies used EHRs as a source of variables for the predictive models. The majority of patients were male and over 60 years old. Table 2 shows the detailed characteristics of the included studies and patients.

**Table 2:** Study characteristics EHR: Electronic health record; ICUs: Intensive care units; IQR: Interquartile range; MAP: Mean arterial pressure; ML: Machine learning; SBP: Systolic blood pressure.

Study ID	Country	Sample size	Study design	Population	Population characteristics		Criteria of CS	Methods of data collection
					Age Mean ± SD	Male N (%)		
Rahman et al. 2022 [19]	USA	24,461	A retrospective cohort	Patients were hospitalized with acute decompensated heart failure.	67.6 ± 15.6	12445 (50.9)	Hypotension (systolic blood pressure ≤ 90 mm Hg or mean arterial pressure ≤ 65 mm Hg) that required inotropic therapy (dopamine, dobutamine, milrinone, or norepinephrine) or mechanical circulatory support and evidence of end organ failure due to a cardiac cause.	By continuously monitoring the patient's EHRs, ML has been developed to identify patients who are at high risk for CS during their hospital stay. This is done by using vital signs, lab values, and medication administrations recorded in the EHRs.
Pinevich et al. 2021 [20]	USA	5,384	A prospective cohort	Patients aged 18 years and older who were admitted to six Mayo Clinic ICUs and five progressive care units. ICUs included cardiac surgery, CV surgical/transplant, medical cardiac, medical, neurology, and trauma/general surgery ICUs. Progressive care units included medical/interventional cardiology, ischemic/comprehensive cardiology, heart failure/valve structural cardiology, CV progressive care, and CV surgical/transplant PCUs.	median (IQR) = 67.9 (56.4-77.6)	2,846 (59.2)	Persistent hypotension defined as SBP < 90 mmHg or MAP 30 mmHg below baseline or requirement of vasopressors to achieve a SBP ≥ 90 mmHg; and (2) signs of impaired organ perfusion (cool extremities, oliguria, and/or alteration in mental status, increased arterial lactate > 2 mmol/L).	Within a 2-hour interval, the ML recorded the patient's vital signs from EHR and ran the algorithm in real time.
Chang et al. 2022 [17]	USA	271	A retrospective cohort	Hospitalized patients who are at risk for CS development, including (AMI and CH).	NA	NA	NA	A machine learning model based on the XGBoost (XGB) algorithm, which runs automatically on patient data from the EHR.
Bai et al. 2021 [21]	China	684	A prospective cohort	AMI patients from the Hospital of Zunyi Medical University.	mean (IQR) = 64.0 (54.0, 73.0)	512 (75)	SBP ≤ 90 mmHg for more than 30 minutes following the exclusion of hypovolemia, with clinical evidence of hypoperfusion (cool extremities or a urine output of < 30 mL/h and a heart rate ≥ 60 beats/min) or the requirement for mechanical left ventricular support to correct the condition	Variables were collected from the patient’s medical records.
Bohm et al. 2022 [16]	USA	3,232	A retrospective cohort	Patients from the critical care units of the Beth Israel Deaconess Medical Center database suffering from acute coronary syndrome are undergoing cardiac catheterizations.	NA	NA	NA	MIMIC dataset (Medical Information Mart for Intensive Care), which includes over 50,000 EHR of patients admitted to critical care units at Beth Israel Deaconess Medical Center in Boston, MA, the USA, from 2001 to 2012.
Jajcay et al. 2023 [18]	USA	2,253	A retrospective cohort	Patients from the critical care units of the Beth Israel Deaconess Medical Center database suffering from acute coronary syndrome are undergoing cardiac catheterizations.	71.0 ± 11.997	1406 (62.4)	NA	MIMIC dataset (Medical Information Mart for Intensive Care), which includes over 50,000 EHR of patients admitted to critical care units at Beth Israel Deaconess Medical Center in Boston, MA, the USA, from 2001 to 2012.

Findings

Meta-Analysis Findings

**Figure 2:**
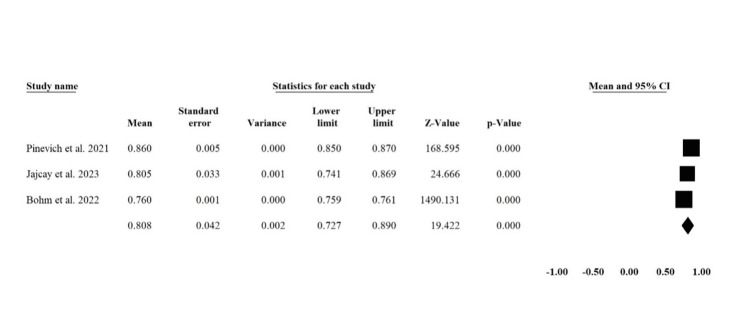
Forest Plot of Pooled Mean AUC for ML Predictive Models This plot represents the combined average Area Under the Curve (AUC) values of machine learning predictive models as reported in three studies: Pinevich et al. 2021 [20], Jajcay et al. 2023 [18], and Bohm et al. 2022 [16].

Area under the curve (AUC):** **Three studies reported [16,18,20] the AUC in a way to be combined in a meta-analysis with a total sample size of 10,869. The pooled mean of AUC was 0.808 (95% CI: 0.727, 0.890) (Figure 2). There was significant heterogeneity across studies (I2 = 99.4%, P < 0.00001), which could be resolved by exclusion of Pinevich et al. 2021 [20] (I2 = 47%, P = 0.168) with the pooled analysis of 0.76 (95% CI: 0.759, 0.761).

Systematic Review Findings

Area under the curve (AUC): The six included studies reported the AUC of the ML predictive models. The AUC ranged between 0.77 and 0.91 in the included studies. Rahman et al. 2022 [19] reported that the AUC of the ML model applied to 24,461 hospitalized acutely decompensated HF patients was 0.77. Chang et al. (2022) [17] recruited 247 patients with congestive heart failure (CHF) and 24 with AMI and found that their ML model AUC was 0.81 in patients with congestive HF and 0.90 in patients with AMI. Bohm et al. 2022 [16] revealed that the AUC of their selected two best-performing algorithms on 3,232 patients hospitalized with AMI was 0.77 and 0.76. Bai et al. 2021 [21] and Jajcay et al. 2023 [18] applied their ML models to 684 and 2,253 AMI patients, respectively, and the AUC was 0.82 and 0.805, respectively. Pinevich et al. (2021) found that the AUC of their ML model applied to 5,384 patients with various backgrounds, including HF and AMI, was 0.86.

Sensitivity, Specificity, and Accuracy

The accuracy of the ML models in predicting CS was reported in four studies [18-21]. It ranged between 0.88 and 0.93. It was 0.88 in CHF hospitalized patients [19], while it was 0.93 and 0.893 in hospitalized patients with AMI in Bai et al. 2021 [21] and Jajcay et al. 2023 [18], respectively. Pinevich et al. (2021) reported an accuracy of 0.921.

The sensitivity and specificity of the ML models were reported in two studies [19,20]. ML had 58% sensitivity and 88% specificity in predicting CS in hospitalized patients with CHF [19]. The ML had a sensitivity of 78.6% (95% CI 74.1%-82.7%) and a specificity of 93.1% (95% CI 92.4%-93.8%) in predicting CS in hospitalized patients in ICUs with various underlying causes, including HF and AMI [20]. 

Predictive Variables

The predictive variables that had the most impact on predicting CS is shown in Table 3. Age and blood pressure were the most reported variables in the six studies, followed by heart rate, oxygen saturation, and blood glucose in four studies. 

**Table 3:** Predictor variables that had the most impact in predicting CS. AMI: Acute myocardial infarction; CHF: Congestive heart failure; ●: indicate that the corresponding study reported this factor as one of the most important factors in predicting CS.

Study ID	The underlying cause	Age	Systolic and pulse pressure	Heart rate	Oxygen saturation	Glucose level	Temperature	Male gender	Immature granulocytes	Respiratory rate	White blood cell count	Troponin level
Rahman et al. 2022 [19]	CHF	●	●	●	●		●			●	●	
Pinevich et al. 2021 [20]	CHF and AMI	●	●		●	●	●	●	●			●
Chang et al. 2022 [17]	CHF and AMI	●	●			●	●	●	●			●
Bai et al. 2021 [21]	AMI	●	●	●							●	
Bohm et al. 2022 [16]	AMI	●	●	●	●	●				●		
Jajcay et al. 2023 [18]	AMI	●	●	●	●	●		●				

Discussion

Predictive medicine holds great promise for improving patient outcomes while lowering costs in the long run [22,23]. We found that AI, using ML predictive models, can accurately predict CS development in patients with AMI or HF. Furthermore, the AUC of ML models demonstrated the great accuracy of these models. We discovered that the most critical variables required by ML models to obtain their outcomes were those that are routinely evaluated in clinical treatment without any additional resources or burden, such as age, blood pressure, heart rate, oxygen saturation, and blood glucose.

The AUC value describes the relationship between the sensitivity and specificity of the model for the prediction of CS [14]. It plots the true positive rates against the false positive rates. Its score ranges from 0 to 1, with 1 being the most accurate for the prediction, with all cases being true positives and no false positives [14]. A grading system was created to describe how well the predictive models are suited for clinical use based on their AUC values [24]. Based on this grading system, our meta-analysis revealed that ML models are good for clinical applications. However, the AUC in the included studies ranged from fair to excellent, which is consistent with the significant heterogeneity found in our meta-analysis. This can be attributed to the different underlying causes, which is evident by the homogeneity observed in the meta-analysis after including only patients with AMI. However, due to limited data, this should be interrupted with caution.

CS can manifest gradually and with nonspecific symptoms, and clinicians will have to rely on regularly monitoring patient data in their medical records using traditional non-AI risk prediction tools, which use vital signs and laboratory data obtained on hospital admission to identify patients at high risk for the development of CS [25]. As these tools are not automated and necessitate ongoing score calculation, adding to the clinicians' workload, this presents a significant challenge for medical professionals. Rahman et al. 2022 [19] managed to develop an AI algorithm that can continuously monitor the hospitalized patients’ vital signs, lab values, and medication administrations, all of which are recorded during routine care in the background. The algorithm could identify patients with a high risk of developing CS who had 10.2 times (95% CI, 6.1-17.2) higher prevalence of CS than those identified by the algorithm as being at low risk. Furthermore, the algorithm could identify high-risk patients 1.7 days sooner than their clinicians' diagnosis of CS. This time period was enough for more than half of the patients to take early preventive measures. AI may thus enable the early identification of high-risk patients and reduce the workload on clinicians, providing physicians with the opportunity to initiate tailored treatment programs, which may prevent future decompensation.

Our findings are consistent with previous studies on the use of ML models in patients with other critical conditions. Mao et al. (2018) [26] found that the ML algorithm performed better than the conventional scoring system and could predict sepsis, severe sepsis, and septic shock with an AUC value of 0.96 using just six vital signs. Brown et al. (2016) [27], using mean blood pressure, temperature, age, heart rate, and white blood cell count, developed a ML model that could detect sepsis early in the emergency department with an AUC value of 0.95. The ML model developed by Hyland et al. 2020 [28] had an AUC value of 0.94 and could detect 90% of circulatory failure events in the ICU, with 82% of cases detected 2 hours in advance.

However, there are still problems that need to be resolved before ML models can be widely used. Robust data processing is definitely becoming more and more important since the creation of predictive algorithms depends on the study of large amounts of data, such as EHRs. However, no dataset is flawless, and incomplete and missing data are inevitable, even with great care and attention to preparation, data collection, and processing [29]. The reliability of the outcomes of these models may be impacted by insufficient handling of missing values, as it can introduce bias in the patient's outcomes [29]. Missing values are commonly encountered in the literature and are usually handled inappropriately in statistical analyses [30]. As a result, additional efforts and research are needed to focus on effectively managing the missing data to produce reliable predictive models.

Limitations

Our study has several limitations. Due to limited data in the literature, we included both AMI and CHF patients, which may have resulted in heterogeneity in our findings. To address this, we ran a sensitivity analysis. Furthermore, the included studies had various sample sizes ranging from 684 to 5,384, which may have contributed to the heterogeneity. All but one of the included studies were from the USA, which may restrict the findings' generalizability. 

## Conclusions

Our findings support the application of AI, namely ML predictive models, in the early prediction of CS in patients with AMI or HF. The ML models predicted CS early with high accuracy using only normally available patient data, such as demographics, examinations, and laboratory variables. However, further high-quality randomized clinical trials are needed to confirm our findings. Furthermore, efforts should be made in the process of data collection, preparation, and managing the missing values.
